# *Oliveria decumbens*, a Bioactive Essential Oil: Chemical Composition and Biological Activities

**Published:** 2019

**Authors:** Mahdieh Eftekhari, Mohammad Reza Shams Ardekani, Mohsen Amin, Farideh Attar, Tahmineh Akbarzadeh, Maliheh Safavi, Elahe Karimpour-razkenari, Mohsen Amini, Murray Isman, Mahnaz Khanavi

**Affiliations:** a *Department of Pharmacognosy, Faculty of Pharmacy and Persian Medicine and Pharmacy Research Center, Tehran University of Medical Sciences, Tehran, Iran. *; b *Department of Drug and Food Control, Faculty of Pharmacy, Tehran University of Medical Sciences, Tehran, Iran. *; c *Department of Biology, Faculty of Sciences, University of Tehran, Tehran, Iran.*; d *Department of Medicinal Chemistry, Faculty of Pharmacy, Tehran University of Medical Sciences, Tehran, Iran. *; e *Department of Biotechnology, Iranian Research Organization for Science and Technology, Tehran, Iran.*; f *Persian Medicine and Pharmacy Research Center, Tehran University of Medical Sciences, Tehran, Iran.*; g *Faculty of Land and Food Systems, University of British Columbia, Vancouver, BC, Canada.*

**Keywords:** Oliveria decumbens, Antibacterial, Anti-Helicobacter pylori, Insecticide, Acetylcholinesterase, Cytotoxic

## Abstract

*Oliveria decumbens* is an aromatic plant traditionally used for treatment of infections and gastrointestinal diseases. In the present study, the volatile oil of the plant was obtained by hydrodistillation and analyzed by GC-MS. In addition, antibacterial and anti-*Helicobacter pylori* activities of this essential oil were determined using disc diffusion and agar dilution methods, respectively. Insecticidal activity was assessed through topical and fumigation application of the essential oil to cabbage looper larvae. Acetylcholinesterase (AChE) inhibition by the essential oil was examined using Ellman’s method. Furthermore, its cytotoxic potential against three different cancer cell lines was assessed using the MTT assay. The phenolic monoterpenoids, thymol (38.79%), and carvacrol (36.30%) were identified as major constituents of the essential oil. We observed significant antibacterial activity of the essential oil against *H. pylori* (MIC=20.4 µg /mL) as well as other tested bacteria, except for *Pseudomonas aeruginosa*. *O. decumbens* essential oil showed significant toxicity to cabbage looper larvae with LD_50 _value of 52.1 µg /larva following topical and fumigant administration. *O. decumbens* essential oil was considerably inhibitory to acetylcholinesterase activity (IC50 = 0.117 µg/mL). Cytotoxic assay of the volatile oil resulted in IC50 = 0.065, 0.104, and 0.141 μg/mL for MCF-7, T47D and MDA- MB-231 cell lines, respectively. According to our data, this species with high concentrations of thymol and carvacrol could be considered as a natural source for pharmaceutical products.

## Introduction

*Oliveria decumbens* Vent. (*Carum orientalum *(DC) Hiroe) is an annual herb in the family Apiaceae which is distributed in south-east Anatolia, Syria, Iraq, and Iran (1). In Iran, the plant grows in limited areas of the south and the west, and known as Moshkoorak, Den or Denak (2). This herb has been used in the traditional or folk medicine treatment of a broad spectrum of disorders such as indigestion, diarrhea, abdominal pains, fever, and infections (3). Our studies of traditional Persian medical resources indicate that this herb as “Shavasara or Moshk choopan” has been used as a liver and heart tonic as well as a CNS stimulant (4-6). 

In addition, several herbal essential oils and their constituents have been reported to have insecticidal effects supporting their use as environment friendly insecticides. The cabbage looper, *Trichoplusia ni *(Lepidoptera: Noctuidae), is an important agricultural pest native to the Americas but now also found in Asia and Africa. This pest threatens numerous cruciferous, and other vegetable crops, and serves as a robust model for insecticidal assessments (7). 

Previous studies of *O. decumbens* essential oil reported thymol and carvacrol as the main constituents (3,8). Moreover, the considerable antioxidant and antibacterial effects of the essential oil have been ascribed to the presence of phenolic compounds (9). Thymol and carvacrol exhibit a wide spectrum of biological effects such as antibacterial (10), anti-*Helicobacter pylori* (11), and cytotoxic activities (12). Both of components were shown to possess a strong toxic effect against the cabbage looper in a previous study (7). These compounds also showed considerable anticholinesterase activity although the relationship to their insecticidal effect is controversial (13,14). Traditional background and pharmacological effects of major essential oil compounds of* O. decumbens* encouraged us to investigate its various biological activities, including antibacterial, anti-*Helicobacter pylori*, insecticidal, anti-cholinesterase and cytotoxic activities. Moreover, our sample of *O. decumbens *essential oil, from plants collected at Kohgiluyeh va Boyer Ahmad Province, Iran was analyzed by GC/MS.

## Experimental

*Plant Collection*

**Figure 1:**
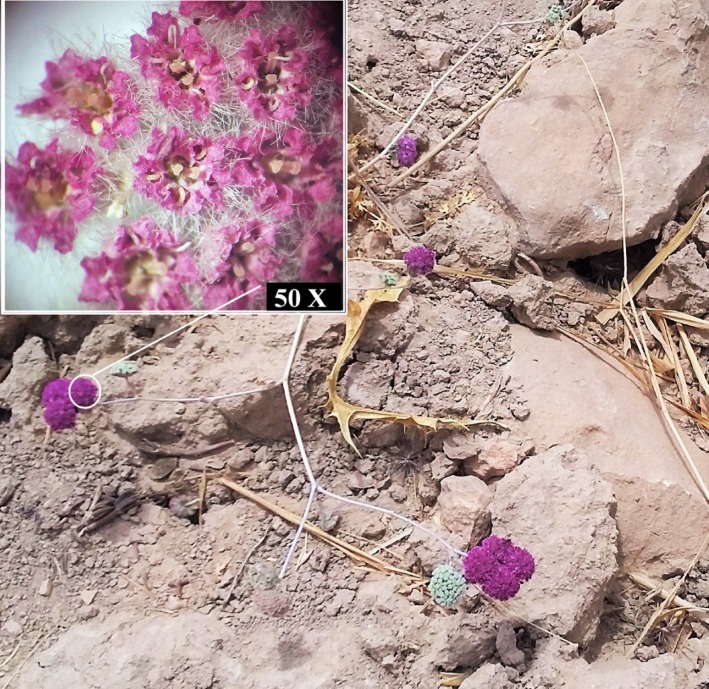
Flowering aerial parts of Oliveria decumbens Vent.

The flowering aerial parts of *O. decumbens *(Figure 1) were collected from Choram, Kohgiluyeh va Boyer Ahmad province located in southwest of Iran (30˚78̍ N, 50˚70̍ E, at 740 m altitude above sea level) on 1 June 2014. A specimen of the plant was identified by Prof. F. Attar and deposited in the Central Herbarium of Tehran University (No.451500 TUH).

*Isolation of the essential oil*

The aerial parts of *O. decumbens *were dried in the shade, ground, and subjected to hydro-distillation using a Clevenger-type apparatus for 5 h. The isolated essential oil was dried over anhydrous Na_2_SO_4_.The density was determined then stored in a sealed dark vial at 4 °C until use (15). 

**Table 1:** *Oliveria decumbens *(aerial parts) essential oil: Chemical composition

**No**	**Compound**	**RT**	**Percentage**	**RRI**
1	α-Pinene	8.63	0.06	932.8
2	β -Pinene	10.17	0.80	975.08
3	β-Myrcene	10.75	0.10	991.32
4	p-Cymene	11.92	7.60	1025.1
5	Limonene	12.04	1.26	1028.6
6	γ-Terpinene	13.10	4.71	1059.8
7	cis-Limonene oxide	15.54	0.07	1134.4
8	trans-Limonene oxide	15.68	0.06	1138.8
9	Unknown	16.57	0.47	1167.5
10	Unknown	16.62	0.72	1169.1
11	4-Terpineol	16.91	0.06	1178.5
12	Unknown	19.37	0.05	1262.4
13	Thymol	20.23	38.79	1292.2
14	Carvacrol	20.43	36.30	1299.2
15	Myristicin	26.28	7.75	1520.0
16	Elemicin	27.05	0.85	1554.0
17	Nonadecane	34.42	0.11	1900
	Total		99.76	
	Monoterpene hydrocarbons		14.53	
	Oxygenated monoterpenes		75.28	
	Phenylpropenes		8.6	
	Other		1.35	

RRI: relative retention indices on DB-5,

RT: Retention times

**Table 2:** Inhibition zone (mm) of *Oliveria decumbens *essential oil and antibiotic discs against some pathogenic bacteria by the disc diffusion method

**Concentrations of essential oil (µg/mL) and antibiotic discs**								
**Bacterial species**	**0.5**	**0.765**	**1.53**	**3.825**	**15**	**20.4**	**Ciprofloxacin 5 µg/disc**	**Penicillin 10µg/disc**
*Staphylococcus aureus*	NZ	6.5mm	7mm	10mm	12 mm	20 mm	24mm	24mm
*Staphylococcus epidermidis*	NZ	NZ	NZ	NZ	10 mm	15 mm	36mm	26mm
*Escherichia coli*	NZ	NZ	7 mm	8 mm	10 mm	14 mm	33mm	10mm
*Pseudomonas aeruginosa*	NZ	NZ	NZ	NZ	NZ	NZ	32mm	NZ

NZ = No inhibition zone was observed.

**Table 3:** *Oliveria decumbens *essential oil and thymol: Minimum inhibitory concentrations *(*MIC) for the growth of *Helicobacter pylori *by the agar dilution method

**Compound**	**MIC (µg /mL)**
Essential oil	20.4
Thymol	150
Amoxicillin	50

**Table 4:** Toxicity of *Oliveria decumbens *essential oil and main constituents against *Trichoplusia ni *via topical application

**Constituents**	a **LD (µg larva** **1** **)** **50**	b **95% CL**	b **95% CL**
*O. decumbens *oil	52.1	38.2-101.7	93.4-221.2
Artificial Mixture^c^	30.8	21.1-42.8	46.6-70.1
Thymol	50.1	39.8-71.1	89.4-146.1
Carvacrol	68.8	51.2-83.1	171.0-236.9
Myristicin	32.7	28.1-38.5	41.7-73.4
ρ-Cymene	202.8	165.3-329.1	342.1-701.5

N = 10 for each test (3 replicates)

a LC , LC = Lethal concentration of larval by 50%, 95% relative to the control group

b 95% CL= denotes 95% confidence limit

c mixture of the essential oil constituents at their natural ratio based on GC/MS analysis

**Table 5:** Toxicity of the main constituents of *Oliveria decumbens *against *T. ni *via fumigation

**Constituents**	a **LD (µg/mL)** **50**	b **95% CL**	b **95% CL**
*O. decumbens *essential oil	93.6	68.1-164.1	257.6-886.4
Artificial Mixture^c^	78.8	65.8-100.9	139.8-408.7
Thymol	365.4	194.7-678.2	---
Carvacrol	222.1	123.9-577.8	---
Myristicin	382.4	220.2-739.3	---
ρ-Cymene	97.9	21.9-215.6	174.4-430.7

N = 10 for each test (3 replicates)

a LC = Lethal concentration of larval by 50% relative to the control group

b 95% CL= denotes 95% confidence limit

c mixture of the essential oil constituents at their natural ratio based on GC/MS analysis

**Table 6:** *Oliveria decumbens *essential oil: Acetyl- and butyrylcholinesterase inhibitory activities

**Sample**	**Acetylcholinesterase IC** **50**	**Butyrylcholinesterase IC** **50**
**Essential oil**	0.117± 0.049 (µg/mL)	>0.5* µg/m
**Tacrine**	0.0095± 0.0022(µg/mL)	0.0020±0.0008 (µg/mL)

* Higher concentrations were impossible owing to turbidity in samples.

**Table 7:** *Oliveria decumbens *essential oil: Cytotoxic activity on breast cancer cell lines

**Sample**	**MCF-7 IC** **50 ** **μg/mL**	**T-47D IC** **50 ** **μg/mL**	**MDA-MB-231 IC** **50 ** **μg/mL**
Essential oil	0.065 ± 0.016	0.104 ± 0.056	0.141± 0.036
Carvacrol	0.019 (36)	---	15.02 (37)
Thymol	0.072 (36)	---	---
Etoposide	16.082± 0.095	18.286 ± 0.064	19.639 ± 0.149

*Gas chromatography-mass spectroscopy*

The essential oil was analyzed using an Agilent gas chromatograph equipped with a flame ionization detector (FID). A 30 m DB-5 capillary column was used with helium at a flow rate of 1 mL/minute as the carrier gas. The column temperature program was as follows: 5 min isothermal at 50 °C, increased to 280 °C at a rate of 3 °C min^–1^ and finally held at this temperature for 10 min. Injector and detector temperatures were 280 °C and 300 °C, respectively. Injection volume was 1.0 μL (split ratio, 1:25). 

Mass spectrometry was accomplished using a Thermo Quest instrument with a quadrupole detector under the same conditions as for chromatography mentioned above. Mass spectra were analyzed at 70 eV ionization energy. Retention indices of peaks were computed using retention times of an n-alkane ladder that was injected after the essential oil. Compounds were identified by comparison of their mass spectra and their retention indices with those reported in the literature or computer library (16,17).

*Biological activities*

*Antibacterial activity*

Antibacterial effect of the essential oil was assessed using a disc diffusion method against two Gram-positive bacteria (*Staphylococcus aureus *and *Staphylococcus epidermidis*) and two Gram-negative bacteria (*Escherichia coli *and* Pseudomonas* *aeruginosa*). Overnight culture of bacteria was used to prepare a bacterial inoculum in sterile normal saline (0.9% NaCl). The inoculum was standardized to the turbidity of 0.5 McFarland (the density equivalent to 1.5 × 10^8 ^CFU/mL). The surface of Mueller-Hinton (MH) agar plates were covered by bacterial suspension. Different concentrations of the essential oil were prepared in methanol. Sterile blank discs (6.4 mm diameter) containing different concentrations of the essential oil (15 μL) were placed on the agar plates. The mean inhibition zone diameter for each concentration of the essential oil was computed after overnight incubation at 37 °C. The lowest concentration of the essential oil that produced a zone of inhibition was considered as showing “antibacterial activity”, meaning the bacterial species was susceptible. A disc containing methanol alone served as the negative control. Standard discs containing ciprofloxacin (5 μg/disc) and penicillin (10 μg/disc) were applied as positive controls. Each concentration was tested in triplicate (18).

*Anti-Helicobacter pylori activity*

Anti-*Helicobacter pylori *effect of the essential oil was examined by an agar dilution method. *H. pylori *strain RIGLD-HC180 obtained from antral biopsy samples of patients at Taleghani Hospital, Tehran, Iran was used in this study. *H. pylori* was cultured on *Brucella* agar (Merck, Darmstadt, Germany) supplemented with 20% fetal bovine serum (FBS; Gibco, USA). The plates were incubated for 3 days at 37°C in microaerophilic conditions using Microbiology Anaerocult^®^ C bags (Merck, Darmstadt, Germany). Anaerocult^®^ C bags are used for production of an O_2_ depleted and CO_2_- enriched atmosphere in an anaerobic jar. The bacterial suspension was prepared in sterile normal saline and adjusted to 0.5 McFarland standard (1.5 × 10^8 ^CFU/mL). Different concentrations of the essential oil or thymol (Sigma-Aldrich, USA), the major constituent of the essential oil, were prepared in methanol. Serial dilutions of the essential oil or thymol were prepared in *Brucella* agar supplemented with 20% fetal bovine serum. This was done before solidifying the medium at 50 °C. After solidification, 10 μL of bacterial suspension was spread over the plates using a sterile loop. The plates were put into an anaerobic jar under microaerophilic conditions and incubated at 37 °C for 72 h as explained above. All samples were prepared in triplicate. The lowest concentrations of the essential oil or thymol that inhibited visible bacterial growth (containing no colonies) were expressed as minimum inhibitory concentrations (MICs). Plates containing methanol and amoxicillin (Sigma-Aldrich, Germany) (50 μg/mL) were used as negative and positive controls, respectively (19). 

*Insecticidal activity*

Acute toxicity of the essential oil and its main constituents (thymol, carvacrol, ρ-cymene, and myristicin (purity range from 95-99.5%) purchased from Sigma-Aldrich, USA) were evaluated through topical and fumigant tests against cabbage loopers (*Trichoplusia ni)* according to Tak *et al.* 2016 method (20).

*Anti-cholinesterase and anti-butyrylcholinesterase activities*

Anti-acetylcholinesterase (AChE) and anti-butyrylcholinesterase (BuChE; Sigma Aldrich) activities of the essential oil were conducted using the Ellman method with slight modification (21). The essential oil was dissolved in methanol: DMSO (1:1 v/v). Then, serial dilutions of the essential oil were prepared in phosphate-buffered saline (PBS; 100 mM, pH 8.0). In the next step, 25 µL of different dilutions of the oil were added to the mixture of 25 µL of AChE (Sigma Aldrich) or BChE (Sigma Aldrich) solution (0.22 U/mL) and 50 µL PBS. The mixture was incubated at room temperature for 15 min, after which 125 µL of DTNB and acetylthiocholine iodide and butyrylthiocholine iodide as substrates were added. Using a microplate reader (ELX808, BioTek, USA) the absorbance was measured at 412 nm after 15 min. A tube containing all reagents except the test compound was used as a negative control (blank). Tacrine was applied as a positive control. All experiments were conducted in triplicate. The inhibitory effect of a sample was calculated as follows:

Inhibition (%) = ((A_b_-A_s_)/A_b_) × 100

A_b_ and A_s_ are the absorbance of blank and sample respectively. The IC_50_ (the required concentration of the essential oil for 50% inhibition of AChE and BuChE) was calculated by liner regression analysis (22).

*Cytotoxic activity by the MTT assay*

MCF-7, MDA-MB-231, and T47D (human breast cancer cell lines) were purchased from Pasture Institute of Iran, Tehran, Iran. Briefly, the cell lines were cultured in RPMI 1640 medium (PAA, Germany) containing N-Hydroxyethylpiperazone-n-2-Ethanesulfonic Acid (HEPES, Biosera, England), sodium bicarbonate supplemented with 1% antibiotics (100 μg/mL streptomycin and 100 U/mL penicillin (Biosera, England)) and 10% fetal bovine serum (FBS; Gibco, USA). All three cell lines were incubated in air with 5% carbon dioxide at 37 °C. The cytotoxic activity of the essential oil was measured by the MTT (3-[4,5-dimethylthiazole-2-yl]-2,5-diphenyltetrazolium bromide)( Sigma-Aldrich, USA) assay with slight modification (23).

The cell suspensions were seeded into 96-well plates and incubated at 37 °C in air with 5% CO2 overnight. Three different concentrations of *O. decumbens* essential oil were added to wells and incubated for 24 h. The MTT reagent (5 mg/mL) and the media were added per well and incubated for 4 h. Then, the medium of each well was removed and DMSO was added into wells, and absorbance measured at 545 nm using a microplate reader. The wells containing DMSO and etoposide were applied as negative and positive controls. Finally, IC_50_ (the required concentrations of the essential oil for inhibition of 50% of cell growth) values were calculated. All tests were replicated three times (23).

## Results and Discussion

*Chemical composition of the essential oil*

The aerial parts of *O. decumbens* produced 3.2% v/w pale yellow essential oil. As seen in Table 1, seventeen constituents were identified, representing 99.76% of the total weight of the essential oil. Thymol and carvacrol, oxygenated monoterpene compounds, were the major components of the volatile oil (75.09%). Other constituents of the essential oil were monoterpene hydrocarbons (14.53%) and phenylpropenes (8.6%). 

There are several reports on the analysis of *O. decumbens* essential oil obtained from diverse geographic regions. Consistent with our results, most previous reports indicate thymol and carvacrol ­­­­as major constituents of the essential oil (3,24), whereas γ-terpinene and myristicin were identified as major compounds of *O. decumbens* oil collected from Charmahale va Bakhtiary province, Iran (25). In contrast to our data, no carvacrol was found in *O. decumbens* essential oil collected from Lorestan province, Iran (26). This shows that differences in essential oil composition can be due to many factors such as variety of tested plant parts, geographic region and collection time of the plant.

*Biological activity*

*Antibacterial activity*

Antibacterial activity of the essential oil was observed using the disc diffusion method. As shown in Table 2, the essential oil possessed a potent antimicrobial effect against *Staphylococcus aureus*,* Staphylococcus epidermidis* and *Escherichia coli*. However, it did not show any activity against *Pseudomonas aeruginosa *up to 20.4 µg/mL. The strongest effect was exhibited against *S. aureus*, while no inhibition was seen against *P. aeruginosa* in the tested range of concentrations. 

Consistent with other studies, the essential oil examined in our study exhibited considerable antibacterial effect on most of the bacterial strains (3,24,25). This activity is likely due to the presence of the phenolic compounds, thymol and carvacrol, or possibly their synergistic action (10,27). According to our data and other studies, thymol and carvacrol lack antimicrobial activity against *P. aeruginosa* (10). It is also noticeable that *p*-cymene and γ-terpinene, other important constituents of the essential oil, did not exhibit any antibacterial effect when tested individually on the bacterial strains (28).

*Anti-Helicobacter pylori effect*

Anti-*Helicobacter pylori *activity was assessed by an agar dilution method. The anti-*H. pylori *activity of the essential oil and thymol as its major constituent is summarized in Table 3. The essential oil showed significant inhibition of *H. pylori *(MIC = 20.4 µg/mL) whereas thymol inhibited growth of *H. pylori *only at substantially higher concentration (MIC = 150 µg/mL). 

We report significant anti-*Helicobacter pylori* activity of *O. decumbens* essential oil. This is consistent with traditional uses of the herb for treating gastrointestinal disorders (3). A recent report mentioned that carvacrol possessed potent activity against *H. pylori *whereas the presence of thymol decreased the anti-*Helicobacter pylori *effect of carvacrol (11). Consistent with this recent publication, our study exhibited lower activity of thymol against *H. pylori* compared to the intact essential oil (11). Hence, further investigation is required to pinpoint the active principle behind the anti-*Helicobacter pylori* effects of this essential oil.

*Insecticidal activity*

*O. decumbens* essential oil showed significant toxicity to cabbage looper larvae with LD_50 _of 52.1 µg /larva following topical administration (Table 4). Among the main constituents of the essential oil, myristicin showed the strongest toxicity on *T. ni* via topical application with 32.7 µg /larva as LD_50_ values.

As seen in Table 5, toxicity of the essential oil and its main constituents to *T. ni* through fumigation pointed to ρ-cymene as the most potent agent with LD_50_ values of 97.9 µg /ml. The essential oil showed fumigant toxicity to *T. ni* with LD_50_ values of 93.6 µg/ml. 

*O. decumbens *essential oil has significant insecticidal activity on *Trichoplusi ni* via topical and fumigation application. Among its main constituents, myristicin, and *p*-cymene have the most potent inhibitory effects against cabbage looper in topical and fumigation applications, respectively. Thymol and carvacrol as major constituents of the essential oil showed more potent toxicity on *T. ni* when topically applied than via fumigation. According to other studies, thymol and carvacrol have strong toxicity against *Spodoptera litura* and *Trichoplusi ni* (29). Similar to our results, there are reports that myristicin has an insecticidal effect against *T. ni* when applied topically (30,31). Additionally, the essential oil of *Helosciadium nodiflorum* showed strong toxicity against *T. ni* due to the presence of myristicin (30).

*Anticholinesterase and antibutyrylcholinesterase activities*

This study highlighted that the *O. decumbens* essential oil could exert a significant inhibitory effect on acetylcholinesterase. As shown in Table 6, the essential oil inhibited AChE activity at a low concentration (IC_50_= 0.117 ± 0.049 µg/mL), whereas the BuChE inhibitory activity was not observed even at higher concentrations (IC_50_> 0.5^*^ µg/m). Our results showed that *O. decumbens *essential oil containing high proportions of thymol and carvacrol, possessed potent anti-AChE activity. According to previous studies, thymol and carvacrol individually have shown significant inhibitory activity against AChE (13). Moreover, there are several reports of significant anti-AChE activity of essential oils with high content of thymol and carvacrol (32,33). A number of investigations have considered the inhibition of AChE as an important mode-of-action of essential oils in insects, but to date there is no compelling evidence linking AChE inhibition *in-vitro* to insecticidal action *in-vivo* (7,14,34,35). Our findings and those of other studies support the traditional uses of this herb as a CNS stimulant and anti-depressant (4,5).

*Cytotoxic activity*

Cytotoxic activity of the volatile oil is shown in Table 7. *O. decumbens* essential oil has strong inhibitory effects against all three tested cancer cell lines. It is remarkable that the essential oil inhibited proliferation of all three cell lines at lower concentrations compared to the positive control (IC_50 _< 10). 

The presence of phenolic components such as thymol and carvacrol could cause potent cytotoxic activity highlighted in the present investigation (28). Both of these compounds are known to have significant cytotoxicity (12). According to Table 7, cytotoxicity of the essential oil on the MDA-MB-231 cell line is 100 times greater than the activity of carvacrol individually (28), indicating toxicity from some other constituents of the *O. decumbens *essential oil or the synergistic or additive effects of them on this human breast cancer cell line. Another investigation of cytotoxic activity of natural monoterpenes on different cancer cell lines pointed to carvacrol as the most potent cytotoxic agent on many cell lines such as MCF-7 (36). Various results with thymol and carvacrol on different cell lines indicate that further investigation is required to find the mechanism of action for these two compounds individually and in combination on different cell lines.

## Conclusion

*O. decumbens* essential oil with high proportions of thymol and carvacrol could be an effective source of antimicrobial, anti-*Helicobacter pylori*, insecticidal and cytotoxic agents as well as in the treatment of Alzheimer’s disease. Further investigations on biological effects of this plant essential oil and its major constituents are merited to address their mechanisms-of-action *in-vitro* and *in-vivo* to develop a well-understood natural pharmaceutical product.
